# Therapeutic Vaccination in Chronic Hepatitis B: Preclinical Studies in the Woodchuck

**DOI:** 10.1155/2010/817580

**Published:** 2010-09-07

**Authors:** Anna D. Kosinska, Ejuan Zhang, Mengji Lu, Michael Roggendorf

**Affiliations:** Institute for Virology, University Hospital of Essen, University of Duisburg-Essen, Virchowstra*β*e 179, 45122, Essen, Germany

## Abstract

Recommended treatment of chronic hepatitis B with interferon-*α* and/or nucleos(t)ide analogues does not lead to a satisfactory result. Induction of HBV-specific T cells by therapeutic vaccination or immunotherapies may be an innovative strategy to overcome virus persistence. Vaccination with commercially available HBV vaccines in patients did not result in effective control of HBV infection, suggesting that new formulations of therapeutic vaccines are needed. The woodchuck (*Marmota monax*) is a useful preclinical model for developing the new therapeutic approaches in chronic hepadnaviral infections. Several innovative approaches combining antiviral treatments with nucleos(t)ide analogues, DNA vaccines, and protein vaccines were tested in the woodchuck model. In this paper we summarize the available data concerning therapeutic immunization and gene therapy using recombinant viral vectors approaches in woodchucks, which show encouraging results. In addition, we present potential innovations in immunomodulatory strategies to be evaluated in this animal model.

## 1. HBV Infection and Current Treatment Strategies

World Health Organization estimates that about 2 billion people worldwide have been infected with hepatitis B virus (HBV). Since the introduction of preventive vaccination programs against hepatitis B in over 170 countries, the number of new infections is continuously decreasing. Despite the success of prophylactic vaccines, chronic HBV infection is still a global health problem. Over 360 million people are persistently infected with HBV, of whom 1 million die each year from HBV-associated liver cirrhosis or hepatocellular carcinoma (HCC). The outcome of HBV infection varies greatly from person to person. In most of the cases the infection is cleared spontaneously, however, 5%–10% of adults develop chronic infection. By contrast, 40%–90% of children which are born to HBV-infected mothers will progress to develop a persistent liver disease [1]. 

In the recent, years a marked progress has been made in the treatment of chronic hepatitis B. Currently, the two types of antiviral therapies are approved: treatment with pegylated interferon alpha 2a (PEG-IFN*α*) or nucleos(t)ide analogues, such as adefovir, entecavir (ETV), lamivudine, telbivudine, and tenofovir [2–5]. However, the efficacy of those therapies in preventing liver cirrhosis and HCC is still limited. Treatment with PEG-IFN*α* leads to a sustained antiviral response in only one third of patients, regardless of combining the therapy with polymerase inhibitors. On the other hand, the treatment with nucleos(t)ide analogues significantly suppresses HBV replication that leads to a decrease of necroinflammation in the liver. However, those antivirals cannot completely eradicate the virus. After withdrawal of the drug, the rebound of viremia is observed in the majority of patients. Furthermore, the long-term treatment is subsequently associated with the appearance of drug-resistant HBV strains that is often the cause of the therapy failure [6, 7]. Therefore, the new approaches in treating chronic hepatitis B are urgently needed.

## 2. Immunological Control of HBV Infection

It is well documented that an appropriate adaptive immune response is required to efficiently control the HBV infection. T cell-mediated immune response directed against hepatitis B virus antigens is crucial for resolution of the infection [8–12]. HBV-specific CD8^+^ T cells are able to clear HBV-infected hepatocytes by secretion of Th1 antiviral cytokines, such as interferons (IFNs) and tumor necrosis factor alpha (TNF*α*), and direct cytotoxic mechanisms (perforin/granzyme, ligand-ligand induced cell death, e.g., Fas-Fas-L) [12–16]. An early, vigorous, polyclonal, and multispecific cellular immune response against the viral proteins is associated with the clearance of hepatitis B in acutely-infected patients. In contrast, chronic HBV carriers demonstrate weak, transient, or often undetectable CD8^+^ T cell response that correlates with HBV persistence [17–21]. Humoral immune response, especially neutralizing antienvelope antibodies, play a key role in preventing HBV spread to noninfected hepatocytes [20, 22]. 

Recent studies indicate that several mechanisms may be involved in the loss of the function of HBV-specific T cells during chronic hepatitis B. It was shown that high-level viremia negatively influences the virus-specific immune responses. High viral replication in the liver with viral load higher than 10^7^ copies/mL is correlating with hyporesponsiveness of virus-specific CD8^+^ T cells in patients with chronic hepatitis B [23]. Moreover, the prolonged exposure to viral antigens occurring during the chronic viral infections can trigger the T cells to become tolerant and prone to apoptosis. The interaction between programmed death 1 (PD-1) receptor and its ligand PD-L1 (also known as B7-H1) plays an important role to prevent an overreaction of the immune system [24]. Recent studies revealed that inhibitory molecules such as PD-1 and CTLA-4 are markedly upregulated on virus-specific T cells, resulting in exhaustion (e.g., lack of IFN*γ* production and proliferation) [25]. Simultaneously, this mechanism can contribute to the development of the chronic infection by impairment of the effective antiviral response. This hypothesis was previously proven for hepatitis C virus (HCV) [26, 27] and human immunodeficiency virus (HIV) infection in humans [28–30], as well as lymphocytic choriomeningitis virus (LCMV) infection in mice [31, 32], and more recently for HBV [33, 34]. Furthermore, several studies imply that functional defects of antigen presenting cells (APCs), mainly dendritic cells (DCs), may contribute to the impaired T cell response in chronic hepatitis B patients [35–41]. *In vitro* studies showed that DCs isolated from HBV chronic carriers produce lower amount of antiviral cytokines, such as type I interferons and TNF*α*, in comparison to healthy controls [35, 36]. In addition, those DCs are less efficient in T cell activation and stimulation of T cell proliferation [35, 39–41]. The novel report demonstrated that myeloid DCs from chronic HBV patients express increased level of inhibitory PD-L1 molecule and therefore may down regulate functions of HBV-specific T cells [39]. Several investigations underline the significance of CD4^+^ CD25^+^ regulatory T cells in pathogenesis of persistent viral infections [42]. In HCV-and HIV-infected patients, it was shown that regulatory T cells may downregulate HCV- and HIV-specific CD8^+^ and therefore influence the disease progression [43–45]. The role of regulatory T cells in HBV infection is still not clear. Nevertheless, the increased numbers of CD4^+^ CD25^+^ regulatory T cells were detected in the blood and the liver of patients with chronic severe hepatitis B [46]. In addition, the liver itself is an organ with tolerogenic properties that might contribute to the immunological tolerance during chronic HBV infection [47, 48]. Finally, viruses developed the strategies to efficiently evade the host immune response resulting in persistent infections. Viral immune escape due to the mutation of CD4^+^, CD8^+^, and B cell epitopes in a given HLA background have been observed in patients infected with HIV, HCV, and HBV [49–54].

Several studies demonstrate that the treatment with lamivudine alone, or in combination with interleukin-12 (IL-12), result in the restoration of the HBV-specific CD4^+^ and CD8^+^ immune response in chronic HBV-infected individuals. However, the therapeutic effect was not sustained in those patients [55–57].

## 3. Clinical Trials of Therapeutic Immunization

Over 20 years, continuous efforts have been undertaken to develop a therapeutic vaccine for chronic hepatitis B to enhance the virus-specific immune responses and overcome persistent HBV infection [58–71]. 

Numerous clinical trials of therapeutic immunization exploited the conventional prophylactic hepatitis B surface antigen- (HBsAg-) based protein vaccines. These studies demonstrated reductions in viremia, HBeAg/anti-HBe seroconversion, and HBV-specific T cell responses in some patients. However, the anti-viral effect was only transient and did not lead to an effective control of the HBV [58–65]. Combination of the HBsAg protein vaccines with antiviral treatment with lamivudine did not lead to a satisfactory improvement of the therapies [66–68]. 

The strategies designed to specifically stimulate HBV-specific T cell responses were also not successful [69–71]. The lipopeptide-based vaccine containing a single cytotoxic T lymphocyte (CTL) epitope derived from HBV nucleocapsid was able to induce a vigorous primary HBV-specific T cell response in naïve subjects [76]. However, in HBV chronic carriers, the vaccine initiated only poor CTL activity and had no effect on viremia or HBeAg/anti-HBe seroconversion [69]. The DNA vaccine expressing small and middle envelope proteins proved to elicit the HBV-specific cellular immune response in chronic HBV carriers, however, this effect was only transient [70]. 

Yang et al. presented the novel DNA vaccine for treatment of chronic hepatitis and combined the immunizations with lamivudine treatment [71]. The multigene vaccine contains five different plasmids encoding most of HBV antigens and human IL-12 gene as a genetic adjuvant. The combination therapy led to sustained antiviral response in 6 out of 12 HBV chronically infected patients. The responders were able to clear HBeAg and had undetectable viral load at the end of a 52-week follow-up. Those effects were correlating with a detectable T cell response to at least one of the HBV antigens [71]. Nevertheless, further studies are needed to evaluate this strategy on a larger cohort of HBV chronic carriers.

The therapeutic vaccine-based HBsAg complexed with human anti-HBs was proposed by the group of Wen et al. [77]. Immunogenic complexes (ICs) stimulate robust T cell responses by increasing uptake of HBsAg through Fc receptors on APCs and, therefore, modulate HBsAg processing and presentation. It was demonstrated that this vaccine administered to HBeAg-positive patients led to decrease of HBV DNA in serum, HBeAg seroconversion, and development of anti-HBs in part of the subjects [78]. Currently, the IC-based vaccine is the only one that entered phase III of clinical trials in chronic hepatitis B patients [79]. Even though the IC-based vaccine led to antiviral effect, clearance of HBV was not observed in treated patients. It seems that the vaccine alone is not sufficient to achieve the full control over HBV. Therefore, some steps have been undertaken to combine the IC-based vaccine with nucleos(t)ide analogues treatment, (Wen et al., personal communication). The ongoing clinical trial will show whether IC are effective as a therapeutic vaccine in chronic hepatitis B.

## 4. Transgenic Mouse Model for Studies on Therapeutic Immunization

Over the years, various animal models, including chimpanzees, woodchucks, ducks, and HBV transgenic mice, were established for development and evaluation of novel therapeutic strategies. Considering the cost, ethical reasons, and available amount, HBV transgenic mice are the most widely used models. Studies using HBV transgenic mouse models demonstrated that DNA immunization with the expression plasmids encoding different HBV proteins could induce HBV-specific antibodies and stimulate CTL responses. However, the functionality of HBV-specific CTLs induced in transgenic mice may be not fully developed [80–82]. Improvement of DNA vaccination regimen [83] and blockade of PD-1/PD-L1 interaction [34, 84] could enhance functional T cell responses and lead to inhibition of viral replication *in vivo* without causing hepatitis. Apart from the DNA immunizations, the other therapeutic approaches including administration of Toll-like receptor (TLR) ligands, HBV-specific siRNA, and direct activation of APCs were evaluated in HBV transgenic mice [85–87]. Those strategies were able to effectively reduce the HBV replication, and are currently under investigation as combined therapies. Nevertheless, this model has a significant limitation. As the HBV genome is inserted into the mouse chromosome, full HBV life cycle does not take place in the transgenic mice and no liver inflammation can be observed [88]. Thus, the animal models with naturally occurring hepadnaviral infection are required for the long-term evaluation of the therapeutic effect. In comparison to chimpanzees, woodchucks are easily available and affordable.

In this paper we would like to introduce woodchucks as a useful preclinical model for designing of the new therapeutic vaccines in chronic hepadnaviral infections. We will summarize the available data concerning therapeutic immunization approaches in woodchucks and present potential innovations in immunomodulatory strategies that yet to be evaluated on this animal model.

## 5. The Woodchuck as a Preclinical Model for Pathogenesis and Therapy of Chronic Hepatitis B

The Eastern woodchuck (*Marmota monax*) is naturally infected by woodchuck hepatitis virus (WHV). WHV was discovered in 1978 as a virus closely related to HBV [89] and classified as a member of *Hepadnaviridae* family. WHV and HBV show a marked similarity in the virion structure, genomic organization, and the mechanism of replication, but differ in several aspects, for example, regulation of transcription (Table 1) [90]. WHV causes acute self-limiting and chronic infection similar to HBV infection in the pathogenesis and profiles of the virus-specific immune response [91]. This feature of the woodchuck model makes it so significant for investigation of the new therapeutic approaches in chronic hepatitis B.

Experimental infection of neonates or adult woodchucks with WHV reflects the outcome of HBV infection in humans. In adult woodchucks infection with WHV usually leads to the resolution of infection and only 5%–10% of animals will develop the chronic hepatitis. The exposure of woodchuck, neonates to WHV results in development of chronic WHV infection in 60%–75% of the cases [92]. The continuous replication of WHV in the liver during the chronic infection is nearly always associated with development of HCC in the woodchucks [93, 94]. After diagnosis of HCC the survival prognosis of the animals is estimated on about 6 months, like in humans. The common features of HBV- and WHV-induced carcinogenesis give the opportunity to examine the new anti-HCC therapies in the woodchucks [95]. 

For many years, the studies on immunopathogenesis of WHV infection in woodchucks were restricted to determination of humoral immune responses [96]. The lack of appropriate methods to evaluate antigen-specific T cell responses was the serious limitation of this model. 

Proliferation assay for peripheral blood mononuclear cells (PBMCs) based on incorporation of [^3^H]-thymidine by cellular DNA, routinely used for human and mouse system, has been ineffective in the woodchuck PBMCs [97, 98]. The failure of this approach is consistent with the fact that woodchuck lymphocytes do not express the thymidine kinase gene (Menne et al., unpublished results). This obstacle had been overcome by usage of the alternative radioactively labeled nucleotide 2[^3^H]-adenine [72]. Development of 2[^3^H]-adenine-based proliferation assay enabled to detect the T-helper lymphocyte responses after stimulation of woodchuck PBMCs with WHV core, surface and X antigens (WHcAg, WHsAg, and WHxAg, resp.) [72, 99]. In addition, using the 2[^3^H]-adenine-based proliferation assay in PBMCs from acutely infected animals, several T-helper epitopes within WHcAg [72] and WHsAg were identified [Menne et al., unpublished results].

Recently established, a novel CD107a degranulation assay for woodchuck PBMCs and splenocytes made a significant breakthrough in studying pathogenesis of hapadnaviral infections in the woodchuck model [73]. Several studies demonstrated that detection of CD107a, as a degranulation marker, is a suitable method for determination of antigen-specific cytotoxic T lymphocytes [100, 101]. The assay enables detection of WHV-specific CTLs basing on their granule-dependent effector function. Recognition of the infected cells by CTLs results in the exposure of CD107a molecule on the CTL surface. In the woodchuck system, CD107a molecule can be stained by cross-reactive antimouse CD107a antibody, what enables the flow cytometric analysis of the woodchuck CTLs.

**Figure 1:**
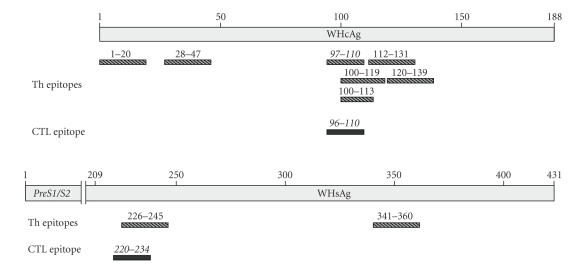
CD4^+^/CD8^+^ T cell epitopes in WHcAg and WHsAg in woodchucks. Immunodominant epitopes' sequences are labelled in italics [72–75].

Introduction of those immunological tools for studying of the T cell response in woodchucks revealed a significant similarity between the pathogenesis of WHV infection in woodchucks and HBV in humans. It was demonstrated that acute self-limiting and resolved WHV infections correlate with robust multifunctional T-helper and cytotoxic T cell responses [72, 73, 99]. Moreover, this efficient cellular immune response to viral antigens results in the liver injury and is necessary for viral clearance. With the novel CD107a degranulation assay, one immunodominant CTL epitope within WHcAg (aa 96–110) [73] and one CTL epitope within the WHsAg (aa 220–234, Frank et al., unpublished results) were characterized (Figure 1). In contrast to self-limiting infection, WHV chronic carriers demonstrate weak or no virus-specific T cell responses against the identified epitopes [72, 73, 99].

The establishment of the assays for monitoring of cellular immune response in woodchucks is of great importance for a reliable evaluation of therapeutic and immunomodulatory strategies for treatment of chronic hepatitis B in the woodchuck model [96, 102, 103].

## 6. Therapeutic Vaccination Approaches in the Woodchuck Model

Recently described advancements in the characterization and monitoring of the woodchuck immune system during the WHV infection, made this animal model particularly useful for development of the immunomodulatory approaches in chronic hepatitis B. The natural occurrence of chronic WHV infection in woodchucks, that is closely related to HBV infection in humans, allows to evaluate the potentially new therapeutic strategies directly in chronic WHV carriers. Up to date, several studies of diverse therapeutic vaccinations have been carried out in woodchucks (Table 2). 

The pioneer investigations based on therapeutic vaccines based on WHV core [96] or surface antigens in combination with a helper peptide FIS [120], or with potent Th1 adjuvants like monophosphoryl lipid A [121] did not lead to satisfactory results. Those experiments proved that vaccinations could induce specific B- and/or T cell responses in chronic WHV carriers. However, this alone was not sufficient to achieve the control of virus replication. 

It is assumed that high level viremia, during the chronic hepatitis B, can inhibit the therapeutic effect of the vaccination. Treatment of chronic HBV patients with lamivudine could transiently restore HBV-specific T cell immune response [55, 56]. Therefore, reduction of viral load by the nucleos(t)ide analogues pretreatment might support the efficacy of immunization to enhance the virus-specific immune responses. This hypothesis was tested in three experimental trials of the combination therapies in chronic WHV carriers.

The first study performed by Hervás-Stubbs et al. was based on lamivudine therapy [108]. Five chronically WHV-infected woodchucks were treated orally with the drug for 23 weeks. At week 10, after decline of WHV DNA by 3–5 logs, three animals were vaccinated with 3 doses of serum-purified WHsAg combined with T-helper FIS peptide derived from sperm whale myoglobin. The vaccination induced T-helper responses against WHV antigens, shifting the cytokine profile from Th2 to Th0/Th1. However, no beneficial effect on WHV viral load and WHsAg levels was observed in comparison to nonimmunized animals. After withdrawal of the lamivudine treatment the values of viremia returned to the pre-treatment levels.

The second trial evaluated the therapy with a very potent antiviral drug: clevudine (previously called L-FMAU) combined with a WHsAg-based immunization [74, 109, 110]. A large cohort of thirty 1-2-year-old chronically WHV-infected woodchucks was enrolled in the study. Half of the animals were orally treated with clevudine (10 mg/kg/day) for 32 weeks; the other 15 woodchucks received placebo. After withdrawal of clevudine treatment, 8 animals from each group were vaccinated with the four doses of formalin inactivated alum-adsorbed WHsAg and 7 were injected with the saline as a control. Combination of the drug and vaccine therapy resulted in marked reductions WHV DNA (6–8 logs) and WHsAg in serum during the 60-week monitoring period, in contrast to the vaccine only and placebo groups, where both markers remained at high levels. Combination therapy did not enhanced anti-WHs responses beyond those measured for vaccine alone. However, treatment with clevudine and vaccine together led to more sustained and robust lymphoproliferative responses to WHsAg and additionally to WHcAg, WHeAg, and WHxAg. Moreover, combination therapy delayed the onset of the liver disease and prevented HCC development in up to 38% of treated chronic WHV carriers in the long-term follow-up study [111]. 

Recently, a novel therapeutic approach for treatment of chronic hepatitis B in a woodchuck model was described. The therapy combined the antiviral treatment with immunization with plasmid DNA and antigen-antibody immunogenic complex vaccines together [112]. DNA vaccines are considered to stimulate both humoral and cellular immune response, polarizing T cells in the direction of Th1 response [122]. Immunization of the naïve woodchucks with the plasmids encoding WHV core and preS2/S genes (pWHcIm and pWHsIm, resp.) induced the lymphoproliferative responses against the antigens and provided a protection against WHV challenge [123]. In addition, the DNA vaccine expressing HBsAg proved to elicit the vigorous T cell responses in chronic HBV carriers, however, this effect was only transient [70]. The HBsAg/anti-HBs IC vaccine is currently under the investigation in chronic HBV patients [77–79]. 

To evaluate the efficacy of previously mentioned immunotherapy in woodchucks, firstly 10 chronic WHV carriers were treated with 15 mg of lamivudine, daily for 21 weeks. At week 10, four animals were pretreated with cardiotoxin and then received three immunizations with DNA vaccine containing three plasmids expressing WHsAg, WHcAg, and woodchuck IFN*γ* (pWHsIm, pWHcIm and pWIFN, resp.). Simultaneously, the other four woodchucks received three doses of the combination of DNA vaccine and WHsAg/anti-WHs immunogenic complex. Two chronic WHV carriers served as lamivudine monotherapy control. Lamivudine treatment resulted in only a slight decrease of WHV DNA levels in the woodchucks serum (0,7 and 0,32 log, resp.). Surprisingly, the DNA vaccination did not lead to any additional therapeutic effect beyond that observed for lamivudine treatment alone. In contrast, the triple combination of antiviral treatment, plasmid DNA encoding WHcAg, WHsAg, and wIFN*γ* and IC vaccines was able to decrease WHV viral load up to 2,9 log and the serum WHsAg up to 92%. Moreover, three of the four treated animals developed anti-WHs antibodies. Nevertheless, these effects were not sustained and all parameters reached the baseline levels shortly after withdrawal of lamivudine treatment. In addition, the vaccination did not induce WHV-specific T cell responses in the majority of woodchucks, even in animals that exhibited virological responses. Significant lymphoproliferative responses against WHV antigens were detected only in one animal after three immunizations with DNA vaccine [112]. The study demonstrated the benefit of using the combinatory therapy in chronically WHV-infected woodchucks. However, the transient therapeutic effects, suggest that this strategy needs further optimization. 

The results from the previous studies clearly confirm the poor efficacy of the lamivudine therapy in woodchucks [108, 112, 124]. A new strategy evaluated the potency of an entecavir treatment and increased number of immunizations [Lu et al., unpublished results]. Chronically WHV-infected woodchucks were pretreated with the entecavir for 21 weeks; 10 weeks in a daily and 11 weeks in a weekly manner. During the weekly administration of the drug, one group of animals received 6 immunizations with two-plasmid DNA vaccine (pWHsIm and pWHcIm),the second group received combination of DNA vaccine together with purified WHV core and surface antigens, and the third group remained untreated. The entecavir therapy resulted in rapid and significant decrease of the viral load and WHsAg levels in serum of the animals. The effect was especially pronounced in animals that additionally received vaccines. In woodchucks treated only with entecavir, the increase of viremia was observed already during the weekly administration or immediately after withdrawal of the drug. By contrast, in both groups of animals, that were immunized with DNA or DNA/proteins vaccines, the delay before the rebound of WHV replication was significantly prolonged. In addition, entecavir treatment was effective to suppress WHV replication and enhanced the induction of WHV-specific T cell responses. An increased CTL activity was detected in individual woodchucks after DNA or DNA/proteins vaccinations. Moreover, two animals completely eliminated the virus from the blood and were WHV DNA negative in the liver [Lu et al., unpublished results].

Altogether, the results obtained in the woodchuck model concerning combination of nucleot(s)ide therapy and immunization proved the synergistic effect of both therapeutical approaches. The therapeutic effects observed during such therapies were significantly increased and prolonged in comparison to the monotherapy alone. In addition, those therapeutic approaches could stimulate the WHV-specific T cell responses, usually impaired in WHV chronic carriers [72, 73]. A combination of antiviral treatment and vaccination is required for the improvement of virus specific T cell responses. Designing of the future therapeutic approaches should include pretreatment with the potent antiviral drugs, such as entecavir or clevudine, that proved their efficacy in the woodchuck model.

## 7. Therapeutic Immunization Using Recombinant Viral Vectors and Prime-Boost Strategy

Previous results from therapeutic immunization trials on woodchucks, chimpanzees, and humans indicate that the licensed vaccines are not able to boost a functional antiviral T cell response. There is a need to use more potent strategies. Vaccines based on recombinant viruses have gained a great interest because of their ability to stimulate robust humoral and cellular immune responses. Viral vectors were investigated as prophylactic and therapeutic vaccines against many human pathogens such as measles virus, herpes simplex virus (HSV), human papillomavirus (HPV), HIV, and rabies [126–130]. However, the utility of those recombinant vaccines in the treatment of chronic hepatitis B was not yet evaluated. 

Preliminary results obtained from the study in chronically HBV-infected chimpanzees immunized with retroviral vector, based on Moloney murine leukemia virus, encoding HBcAg suggest that further investigation of viral-vector based vaccines should be taken into consideration [131]. In the experiment, one of the three therapeutically immunized chronic carrier chimpanzees cleared the virus and showed HBeAg seroconversion. Significant ALT elevations observed in this animal implicate restoration of HBV-specific cytotoxic and humoral responses without causing fulminant hepatitis. Moreover, the other two chimpanzees demonstrated high anti-HBe titers after the therapy and one of them HBcAg-specific CTLs [131]. This study demonstrates not only the benefit of using the recombinant viral-vectors for treatment of chronic HBV infection in primate model, but also the possible advantage of using core antigen-based therapeutic vaccines. Even though the retroviral vector vaccination was well tolerated in the chimpanzees, several clinical trials suggest that gene therapy with traditional retroviral vectors can lead to oncogenesis [132, 133]. Therefore, the usage of another recombinant virus as a carrier of the proteins could be beneficial.

## 8. Recombinant Adenoviruses as the Vaccines

**Figure 2:**
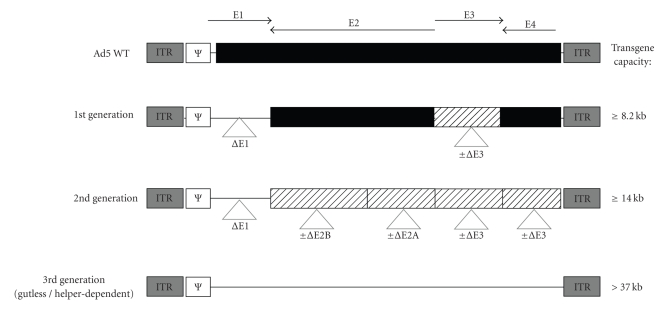
Genome structures of the first, second, and third generation of adenoviral vectors. Wild-type adenoviral sequences are labelled in black. The localization of the early genes (E1–E4) is represented by arrows. Deletion sites are shown as a thin line or as striped boxes for alternative deletions. ITR: inverted terminal repeats; Ψ: packaging signal (modified from: X. Danthinne [125]).

Recombinant adenoviruses have been one of the intensively investigated viral vectors for therapeutic purposes. Development of the novel methods for manipulating of the viral genome resulted in the three generations of the recombinant adenoviruses and with increasing capacity [125] (Figure 2). Several trials imply the usefulness of those vectors in gene therapy of genetic diseases and cancer [134–137]. For many years, the first generation replication-deficient E1 or E1/E3-deleted adenoviral vectors have been explored as the vaccine carriers in prevention of the infectious diseases [138]. Adenoviral vectors have several advantages that can be beneficial for potent therapeutic vaccines.

First of all, adenoviruses are relatively susceptible for genetic modifications and can be easily produced in high titers. After transduction of the cells, adenoviral genome is not integrated into the host DNA and stays in the episomal form. As a result, the risk of the possible activation of the cellular oncogenes is minimal. Adenovirus-based vaccines proved to elicit a vigorous and sustained humoral and T cell responses to the incorporated antigen that is considered to be crucial in clearance of persistent viral diseases [127, 139–141]. The benefit of adenoviral vectors as a vaccine carrier is not only limited to stable delivery of proteins of interest. Several findings on additional immunostimulatory effects, for example, induction of the innate immune response, that originate from the nature of adenoviruses itself, may enhance the vaccine efficacy. Capsid of adenoviruses demonstrates immunostimulatory properties, that is why the coadministration of the adjuvant is usually unnecessary. Those vectors can directly transduce DCs causing their maturation and upregulation of MHC and costimulatory molecules on their surface, thus lead to enhanced antigen presentation. Moreover, it was shown that AdV-transduced DCs are secreting antiviral cytokines, such as IFN*α*, TNF*α*, and IL-6 [142]. Interleukin-6 is one of the most important factors that suppress the function of the regulatory T cells [143, 144]. 

Nevertheless, modified adenoviruses apart from the abovementioned advantages have one serious limitation. Thus far, vectors that were comprehensively examined as the vaccines have been based on the human adenovirus serotype 5 (Ad5) [127]. This serotype is the most common in the human population. Anti-Ad5 neutralizing antibodies are detectable in 45%–90% of adults [145]. The preexisting immunity directed against Ad5 is considered as a main reason of failure in the phase I clinical trial of a protective HIV-1 vaccine. STEP study guided by Merck pharmaceutical concern, based on 3-dose regimen of a trivalent Ad5 vaccine, suggested that the immunization might increase the risk of HIV-1 infection in the subjects with high neutralizing anti-Ad5 titers [146–148]. Moreover, even single immunization may induce immunity to the vector in seronegative individuals. 

The negative effect of the pre-existing or Ad5-induced immunity against the vaccine, mostly when the therapy requires multiple dosages, may be overcome by heterologous prime-boost regimen. The utility of the rare human serotypes (e.g., serotype 35) [149, 150] or recombinant adenoviruses of nonhuman origin has been recently tested [151]. In particular, subsequent priming immunizations with plasmid DNA vaccine followed by a booster vaccination with AdV seem to be a very promising strategy. DNA primeadenovirus boost regimen proved to induce more robust and potent immune response in comparison to plasmid DNA alone and provided protection against the pathogen challenge in several animal models of infectious diseases [149, 152–154]. Furthermore, a clinical trial of multiclade HIV-1 DNA plasmid-Ad5 boost vaccine, HIV-uninfected individuals demonstrated high immunogenicity even in the presence of high anti-Ad5 antibody titer. In addition, the vaccine proved to be well tolerated in the participants of the study [155].

## 9. Improvement of Adenoviral Vectors

Several studies indicate that the transgene expression level can be increased from adenoviral vectors by the presence or insertion of an intron sequences [156–158]. Therefore, we constructed the new recombinant adenoviruses serotype 5 and 35 encoding WHV core protein and containing an intron between promoter and WHcAg gene sequences. Preliminary experiments showed that vaccination with the AdVs containing the intron sequences led to induction of robust cellular and humoral immune responses in mice. Moreover, immunization of the mice in DNA prime-AdV boost manner, using improved vectors, resulted in more vigorous and multispecific T cell responses in comparison to immunization with plasmid DNA alone [Kosinska et al*.,* unpublished results]. 

Immunization of chronically WHV-infected woodchucks with plasmid DNA vaccine in combination with entecavir treatment showed a marked therapeutic effect. Addition of the recombinant adenoviruses to this regimen could be a new, more potent approach in treatment of chronic hepatitis B. We will apply DNA prime-AdV boost approach in WHV chronically infected woodchucks in combination with nucleos(t)ide analogs and evaluate its therapeutic potential.

## 10. Adenoviral Vectors for Gene Transfer Strategies in Treatment of Chronic Hepatitis B

Over the last 20 years, modified adenoviruses have been extensively studied as a vehicle for gene delivery to the liver, because of their high transfection efficiency and their natural tropism for hepatocytes [159, 160]. Moreover, the development of the third generation of adenoviral vectors that lack all viral coding sequences (e.g., helper-dependent adenoviral vectors), resulted in their increased capacity and minimized immunogenicity of the vector allowing long-term transgene expression [161]. High cloning capacity of those vectors enables usage of inducible or tissue-specific promoters and coexpression of multiple therapeutic or immunomodulatory genes [162].

So far, several trials of virus-mediated gene therapy for treatment of chronic hepatitis and HCC were performed in chronically WHV-infected woodchucks and in cell culture systems. Those strategies were mainly based on delivery of antiviral cytokines, such as IFN*α*, IFN*γ*, IL-12 by recombinant adenoviruses, to reduce viral replication or modulate the immune response (Table 3).

Transduction of primary woodchuck hepatocytes from chronic WHV carriers with helper-dependent AdV encoding woodchuck IFN*α* (wIFN*α*) resulted in the reduction of WHV proteins expression *in vitro* [169]. *In vivo *studies on chronically WHV-infected woodchucks, demonstrated that a single injection of 1 × 10^12^ vp of this vector into the liver's portal vein could inhibit WHV replication by 1 log up to 11 weeks after the treatment [163]. The same approach with helper-dependent AdV expressing woodchuck IFN*γ* (wIFN*γ*) did not show any antiviral effect, even though the transduction led to the production of biologically active interferon [163]. Another study combined intravenous delivery wIFN*γ* by recombinant adenoviral vector with nucleos(t)ide analogues therapy. Chronic WHV carriers were treated with clevudine and emtricitabine (FTC), together, for 8 weeks and after the initial drop in viral load one group of animals received additionally two i.v. injections of 3 × 10^10^ PFU of Ad-IFN*γ*. Delivery of wIFN*γ* induced inflammation, caused by T cell infiltration, and increased hepatocyte turnover. However, this effect did not induce additional antiviral outcome in comparison clevudine/emtricitabine biotherapy alone [164]. Similarly, poor therapeutic effect was observed for gene therapy based on both wIFN*γ* and wTNF*α*. Intravenous injection of those recombinant adenoviruses during clevudine treatment led to decrease of replicative intermediates of WHV DNA in the liver, beyond what could be achieved by clevudine alone. Nevertheless, 6 weeks after injection there was no significant difference between the groups of WHV carriers receiving AdV expressing the cytokines or beta-galactosidase as a control [165]. The benefits of using the immunomodulatory genes in this study are difficult to assess, since it was reported that adenovirus infection alone is sufficient to transiently suppress the WHV replication in chronically infected woodchucks [170]. The lack of therapeutic effect by direct delivery of IFN*γ* is consistent with *in vitro* data obtained from persistentlyinfected woodchuck primary hepatocytes. Treatment of the cells with wIFN*γ*, even in the presence of wTNF*α*, was not able to inhibit the WHV replication. Moreover, high concentration of those cytokines resulted in the loss of the cells during the culture [171]. This observation underlines the cytotoxic effect of Th1 cytokines on the woodchuck hepatocytes. Rapid downregulation of the IFN*γ* expression, after transduction of the liver cells with viral vector, could be one of the mechanisms to protect the organism from the potential toxicity of this cytokine in vivo [163]. In addition, several reports indicates that the level of wIFN*γ* and wTNF*α* is higher in the liver of chronic WHV carriers in comparison to naïve animals [172, 173]. Therefore, continuous presence of inflammatory cytokines in the liver during the chronic WHV infection could result in hyporesponsiveness of hepatocytes to such a therapy. 

The novel strategy to treat chronic WHV hepatitis is based on adenovirus-mediated delivery of murine IL-12 (mIL-12) gene into hepatocytes [166]. Interleukin-12 is a proinflammatory cytokine produced naturally by antigen presenting cells. IL-12 stimulates production of IFN*γ* and TNF*α* by T and natural killer (NK) cells and enhances their cytotoxic activity [174]. In the study, mIL-12 gene expression could be regulated by inducible promoter that was responding to progesterone antagonist RU486. Eight chronic WHV carriers received single dose of 2 × 10^10^ i.u. of AdV expressing mIL-12 (HC-Ad/RUmIL-12) by intrahepatic injection at laparotomy. Two weeks after, the expression of mIL-12 was induced by the administration of RU486. The IL-12 treatment resulted in intense and sustained suppression of WHV replication in the liver as well as decreased viral loads in the serum. This effect, however, was visible only in the animals with basal viremia lower than 10^10^ WHV copies per milliliter of serum. Animals, which responded to the therapy, developed a vigorous T cell response to WHcAg, measured by woodchuck IL-2 production, and demonstrated WHeAg and WHsAg seroconversion. Moreover, the FoxP3 levels in the livers of those animals were decreased, while in nonresponder woodchucks FoxP3 values were significantly upregulated [166]. This finding suggests that the intrahepatic expression of IL-12 may inhibit the regulatory T cells in the liver during the chronic WHV infection. Indirect induction of inflammatory cytokines, such as IFN*γ* and TNF*α* by IL-12, seems to be a more efficient strategy in breaking the tolerance to virus antigens than direct delivery of those cytokines. It suggests that probably additional events occur in the liver after AdV-mediated IL-12 transfer that supports the antiviral effects of this therapy.

## 11. Gene Transfer Strategies for the Treatment of Hepatocellular Carcinoma

Adenoviral delivery of genes for cytokines and other immunomodulators is widely used in cancer therapy in the animal tumor models as well as in patients [137, 175–178]. The T cells play an important role not only in defense against the pathogens, but also in antitumor immunity and inhibition of the tumor growth. Interleukin-12 inhibits the angiogenesis and induces a potent antitumoral immune response by stimulation of IFN*γ* secretion. Therefore, IL-12 is a promising candidate for cancer gene therapy [179–183]. Strategy based on recombinant adenoviruses expressing IL-12 demonstrated antitumor effect in the murine models with transplantable HCC [184, 185] and was also evaluated in woodchucks [168]. 

In the study, large (2–5 cm) intrahepatic tumors of 5 woodchucks were injected with a single dose of 1 × 10^9^ PFU AdV expressing IL-12 and B7.1 molecule (AdIL-12/B7.1). The B7.1 molecule (also known as a CD80) is naturally expressed on the professional APCs and provides the synergistic effect in the tumor regression [181, 186, 187]. In 4 out of 5 animals, AdIL-12/B7.1 was delivered by laparotomy into the three HCC nodules and three nodules were injected with a vector expressing GFP as a control. Animals were sacrificed 7–14 days later and the tumor volumes were assessed. On average, treated tumors showed an 80% reduction in the volume whereas the size of the AdGFP-injected nodules increased. Remission of the tumors was associated with CD4^+^ and CD8^+^ T cell infiltration into the tumor tissue and increased local IFN*γ* levels after AdIL-12/B7.1 injection. One of the treated woodchucks received the intratumoral injection by magnetic resonance imaging (MRI) guidance and was monitored for 7 weeks. During this period the tumor size decreased from 8,6 cm^3^ to 0,5 cm^3^ [168]. This observation shows that administration of AdIL-12/B7.1 during MRI guidance, with therapeutic effect similar to laparotomy, could prevent the animals from harmful consequences of the surgery. 

The study proved that the gene therapy based on IL-12 leads may be a promising strategy to treat HCC. By contrast, treatment with AdV encoding herpes simplex thymidine kinase combined with gancclovir administration did not lead to reduction in the tumor size [167]. Nevertheless, the short time of monitoring during the study makes it difficult to evaluate the prolonged antitumoral effect of this approach. 

A recent study presents gene therapy with semliki forest viral vector expressing high levels of murine IL-12 (SFV-enhIL-12) on remission of HCC in chronically WHV-infected woodchucks. In the research, the vector was delivered by surgery into multiple sites of HCC tumors in the liver [75]. A total of nine woodchucks were enrolled in the experiment. Six of the woodchucks, two animals each, received different doses of SFV-enhIL-12: 3 × 10^9^ vp, 6 × 10^9^ vp, and 1,2 × 10^10^ vp, and three animals served as a control and received saline injections. The tumor size was monitored by ultrasound examination for 23 to 24 weeks. In all woodchucks, reduction in tumor volume was observed, however, this effect was transient and dose dependent. Animals treated with the highest dose of SFV-enhIL-12 showed the most spectacular reduction of the tumor size 71% and 80%. Nevertheless, the tumors started to grow between 6 and 14 weeks after the treatment. The antitumoral effect was associated with the induction of the immune response towards the tumor antigens, demonstrated by T cell proliferation assay, upregulation of leukocyte markers expression, and cytokine production, such as IFN*γ*, TNF*α*, IL-6, and IL-12. In addition, the therapy resulted in transient induction of lymphoproliferative responses against WHcAg and WHsAg and led to short-term reduction in WHV viral load [75]. 

The results presented here indicate that viral-mediated gene therapy in treatment of chronic hepatitis B and HCC needs further optimization. However, treatment of the woodchucks with viral vectors allowed to achieve a long-lasting expression of the cytokines and their higher concentration preferably in the liver. Therefore, this strategy is proven to be more effective than an approach based on using of the soluble cytokines. In addition, adenovirus-mediated gene transfer is proven to be a safe and a well-tolerated strategy in the woodchucks.

## 12. Conclusion

The current progress indicates the feasibility of therapeutic approaches for treatment of chronic HBV infection. There is a general agreement that a combination of antiviral treatment and immunomodulation is essential to achieve a sustained control of HBV infection. However, many scientific questions are still not answered. The question how HBV infection leads to defective immune responses to HBV proteins remains to be investigated. This issue is the key to a more rational design of new therapeutic approaches. Recently, HBV proteins were found to suppress host innate responses [188]. It has to be clarified whether an early blockage of innate immune responses may further negatively influence the priming of adaptive immune responses. In addition, different groups reported consistently that TLR2 and TLR4 signalling may be impaired in chronic HBV infection patients [189, 190]. Thus, it is worthy to test whether an enhancement of innate immune responses in chronic carriers is necessary for restoration of specific immune responses. With the increasing number of available vaccine formulation, a more crucial question raised recently: what is the optimal combination of these vaccines. Obviously, it is necessary to test the mutual influences of different types of vaccines to maximize their effects and avoid the negative interference between the vaccines. Finally, the future design of therapeutic vaccines needs to be considered in nonnaïve hosts since patients have undergone other infections. It is yet not possible to foresee how the pre-existing infections and immunological backgrounds will influence the effect of therapeutic vaccines. Understanding these issues will be helpful for the translation of recent progresses for clinical use of therapeutic vaccines.

## Figures and Tables

**Table 1:** Virological and clinical comparison between HBV and WHV.

	HBV	WHV
*Virology*		
Classification	Family: *Hepadnaviridae *	Family: *Hepadnaviridae *
	Genus: *Orthohepadnavirus *	Genus: *Orthohepadnavirus *[91]
Host	Human	Woodchuck (*Marmota monax*)
Structure	40–42 nm spherical; enveloped nucleocapsid; partially double-stranded DNA genome [22]	42–45 nm spherical; enveloped nucleocapsid; partially double-stranded DNA genome [91, 104]
Proteins	Surface glycoproteins (large-L, medium-M, small-S), core protein, “x” protein, “e” antigen, DNA polymerase with reverse-transcriptase activity [22, 105]	The corresponding proteins [91]
Replication strategy	Replication of HBV DNA occurs by reverse transcription of an RNA intermediate within cytoplasmic nucleocapsids [22]	The same mechanism [97]
Genetic diversity	8 major genotypes [105]	1 major genotype (minor sequence differences) [91]
Integration into host chromosome	Yes [22]	Yes, often close to N-*myc* oncogene region [106]

*Clinical course of infection*		
Epidemic	350 million people infected worldwide	Endemic in some woodchuck population in North America
Vertical transmission	The most common: from mother to newborn	Neonatal woodchucks infected by WHV inoculum
	chronicity rate: 45%–90% [20, 105]	chronicity rate: 60%–75% [92]
Horizontal transmission	Transmitted by body fluids, 90% of individuals recover [105]	Adult woodchucks infected by WHV inoculum,
		90%–95% of animals recover [92]
Clinical features of chronic infection	Variable HBV DNA levels: 10^4^–10^12^ copies/mL	WHV DNA levels: 10^9^–10^11^ copies/mL
	Variable HBsAg levels	WHsAg: mean 100–300 ug/mL
	liver transaminases elevation [20, 22, 105]	liver transaminases elevation [92, 107]

*Disease progression*		
Liver cirrhosis	2%–5% in HBeAg-positive patients (genotype dependent) [20]	Not common
Hepatocellular carcinoma	5-year cumulative HCC incidence in patients with cirrhosis: 16% (data in Asia) [20]	Nearly 100% of chronic infected animals have HCC after 3 years [92, 94, 95, 107]
Efficacy of nucleos(t)ide analogues treatment	Effective: entecavir, tenofovir, telbivudine, adefovir, lamivudine [2–5]	Effective: clevudine, telbivudine, entecavir, emtricitabine, tenofovir,
		Less effective: tenofovir, adefovir, lamivudine [74, 108–118]
Development of resistance mutations	Yes [6, 7, 105]	Lamivudine-resistant strains isolated [119]

**Table 2:** Studies on therapeutic vaccinations in the woodchuck model.

Vaccines	Application	Adjuvants	Outcome	Reference
WHcAg	intramuscular		Viral elimination in 1 of 6 animals	Roggendorf et al., 1995 [96]
WHsAg and Th-peptide	intramuscular	Th-peptide	Transient anti-WHs antibody responseTwo woodchucks died	Hervás-Stubbs et al., 1997 [120]
WHsAg and Th-peptide	intramuscular	Th-peptide	No induction of anti-WHs antibodiesDetectable T-cell responses to WHV proteins	Hervás-Stubbs et al., 2001 [108]
WHsAg in combination with clevudine (L-FMAU)	intramuscular	alum	Reduction of serum viral loads and viral replication in liverInduction of anti-WHs and detection of T-cell responses to WHV proteinsDelayed occurrence of HCC	Menne et al., 2000, 2002 [74, 109, 110]
WHsAg	intramuscular	monophosphoryl lipid A	No reduction of serum viral load, Development of antibodies to the preS region of WHsAg	Lu et al., 2003 [121]
Plasmid DNA expressing WHsAg, WHcAg, and woodchuck IFN-*γ* in combination with lamivudine	intramuscular	*∅*	Transient reduction of serum viral loads	Lu et al*.,* 2008 [112]
WHsAg/anti-WHs immunogenic complex and DNA vaccines in combination with lamivudine	intramuscular	*∅*	Transient reduction of serum viral loadsTransient appearance of anti-WHs antibodies and WHcAg-specific T cell response	Lu et al*.,* 2008 [112]
Plasmid DNA encoding WHsAg and WHcAg in combination with entecavir	intramuscular	*∅*	Transient reduction of serum viral loads	Lu et al*.,* [unpublished results]
Plasmid DNA encoding WHsAg and WHcAg in combination with protein WHsAg/WHcAg vaccine in combination with entecavir	intramuscular	*∅*		

**Table 3:** Studies on gene therapy of chronic hepatitis and HCC in the woodchuck model.

Vector	Application	Outcome	>Reference
Helper-dependent AdV expressing woodchuck IFN*α*	Intravenous (portal vein)	Transient inhibition of WHV replication	Fiedler et al., 2004 [163]
Helper-dependent AdV expressing woodchuck IFN*γ*	Intravenous (portal vein)	No effect	Fiedler et al., 2004 [163]
AdV expressing woodchuck IFN*γ* in combination with clevudine (L-FMAU) and emtricitabine (FTC)	intravenous	T-cell infiltration and inflammation in the liver	Jacquard et al*.,* 2004 [164]
		No additional antiviral effect beyond the treatment with the nucleot(s)ide analogues	
AdV expressing woodchuck IFN*γ* and TNF*α* in combination with clevudine (L-FMAU)	intravenous	Transient inhibition of WHV replication	Zhu et al*.,* 2004 [165]
High-capacity AdV expressing murine IL-12 under the control of a liver-specific inducible promoter	intrahepatic (via laparotomy)	Inhibition of WHV replication in the liver and decreased viral load in serum.	Crettaz et al*.,* 2009 [166]
		Induction of anti-WHs antibodies.	
		The effect was observed only in animals with basal viremia lower than 10^10^ copies/mL.	
AdV expressing herpes simplex virus thimidine kinase combined with gancyclovir treatment	intratumoural (via laparotomy)	Necrotic areas in the tumour mass and in the liver.	Bilbao et al*.,* 2000 [167]
		No reduction in tumour volume.	
AdV expressing murine IL-12 and B7.1 molecule	intratumoural (via laparotomy and MRI guidance)	CD4^+^ and CD8^+^T cell infiltration in the liver.	Pützer et al*.,* 2001 [168]
		Reduction in tumour volume.	
Semliki forest viral vector expressing murine IL-12	intratumoural (via laparotomy)	Induction of T cell responses to tumour antigens.	Rodriguez-Madoz et al*.,* 2009 [75]
		Induction T cell responses to WHcAg and WHsAg.	
		Dose-dependent, transient reduction in tumour volume.	
